# Supplementary material for: Pigeon Circovirus over Three Decades of Research: Bibliometrics, Scoping Review, and Perspectives
Source: Viruses. 2022 Jul 8;14(7):1498. doi: 10.3390/v14071498 (PMC9317399; doi:10.3390/v14071498)
Supplement: Supplementary file 1 [file viruses-14-01498-s001.zip › viruses-1799092 resubmitted proof Supplemental Figures.pdf]

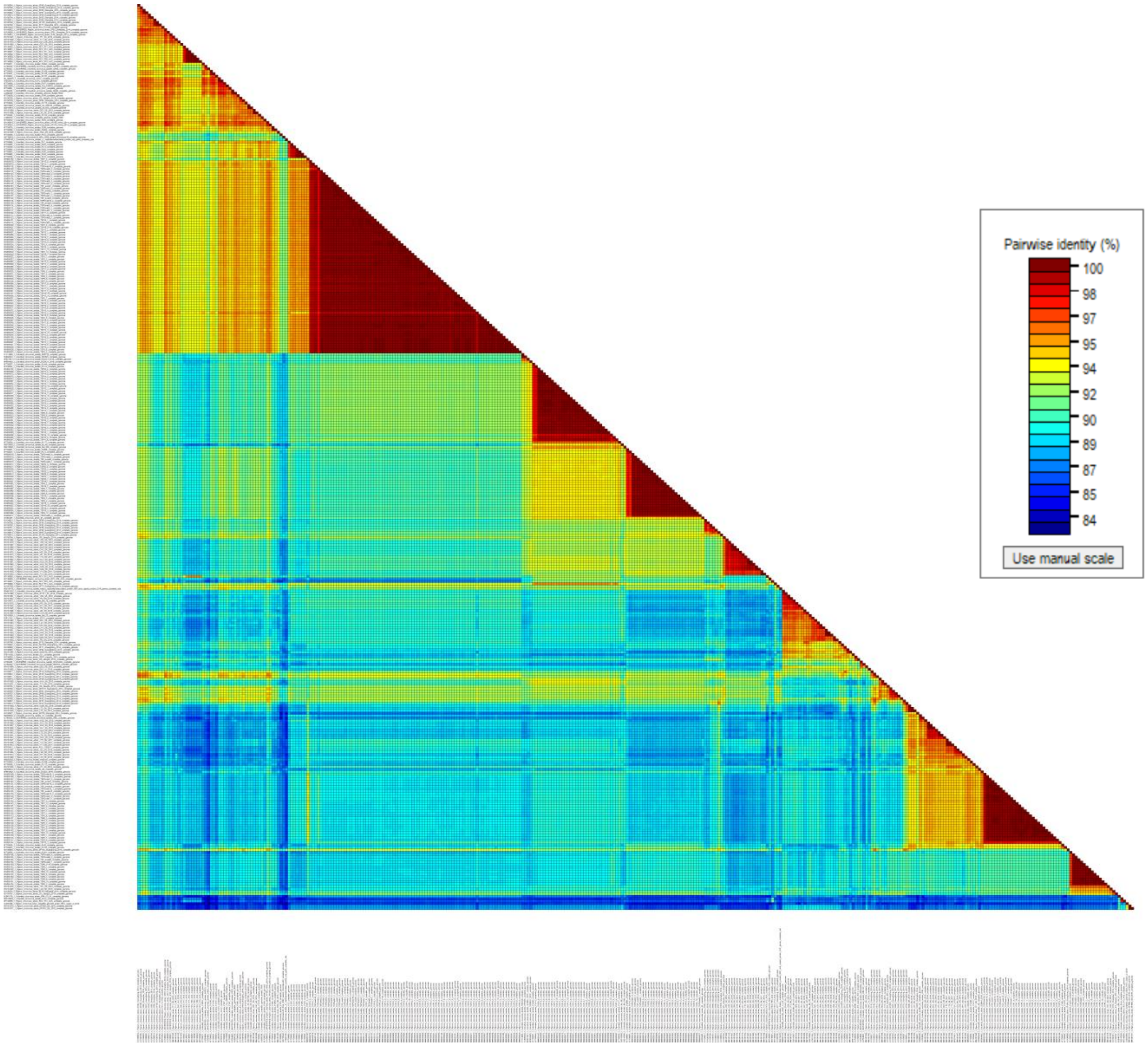

Supplementary Figure S1. Pairwise identity matrix of 371 PiCV whole genome sequences available from NCBI.

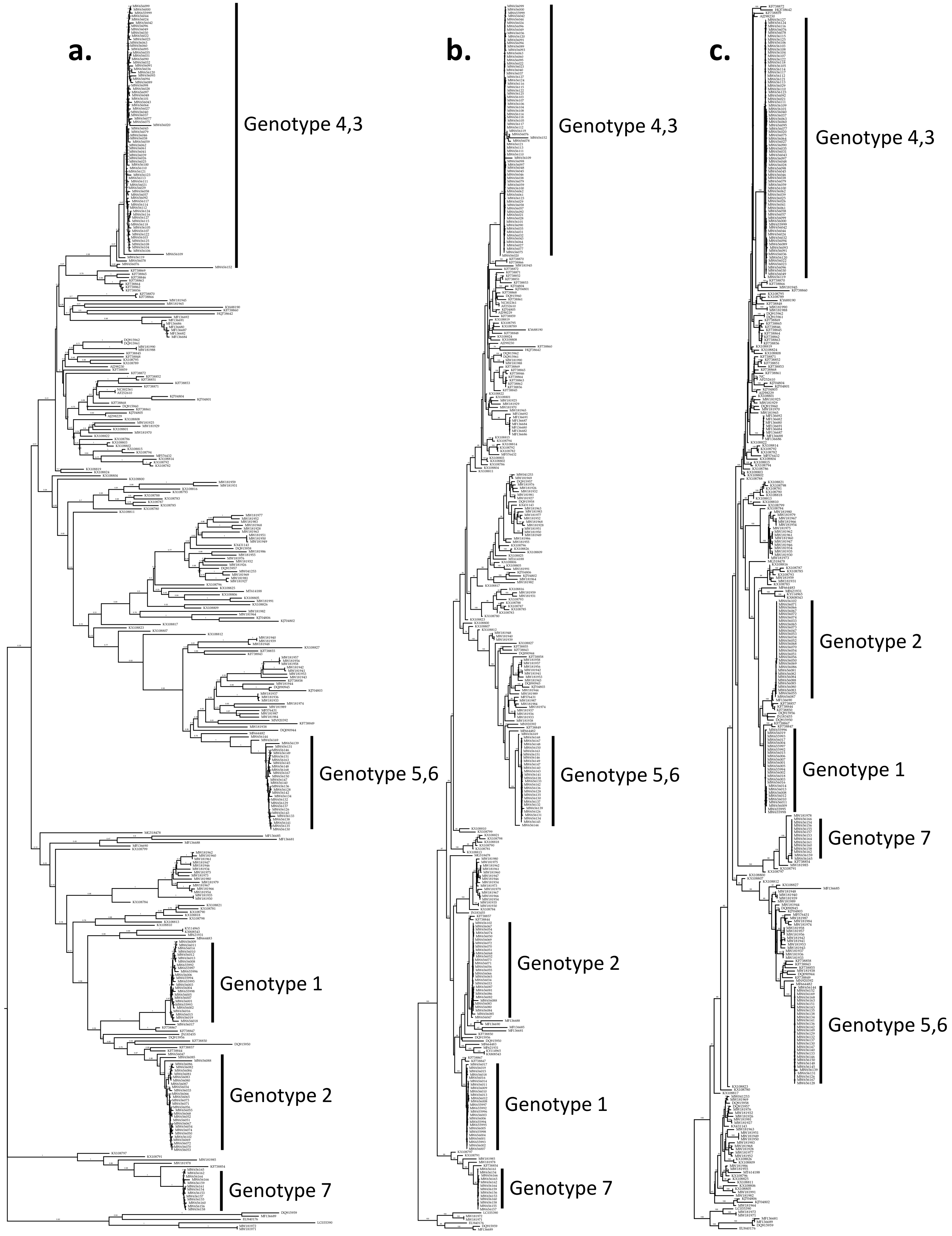

Supplementary Figure S2. Phylogenetic trees of the 371 PiCV whole genome sequences available from NCBI. (A) Neighbor-joining tree and (B) Maximum-Likelihood tree of the 371 whole genome sequences of PiCV, while (C.) is the Maximum-Likelihood tree of the 371 whole genome sequences of PiCV after removing the recombinant regions. Genotypes were named as in Khalifeh et al. (2021) [73].

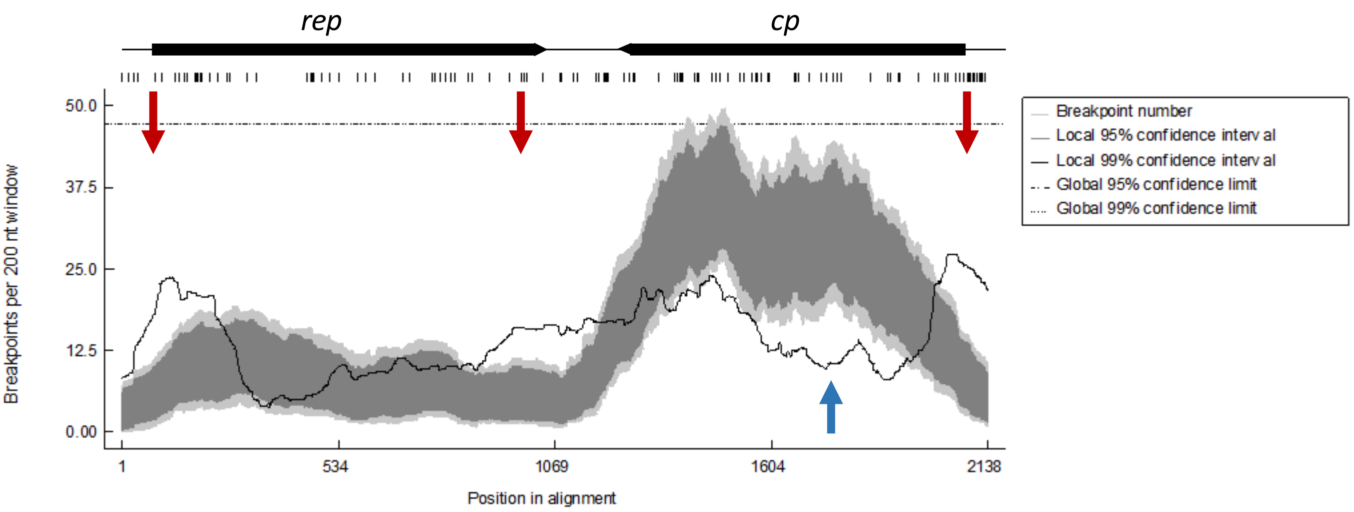

Supplementary Figure S3. Recombination breakpoint analysis of the 371 PiCV whole genome sequences available from NCBI. General hot spots (red) and cold spots (blue) regions were marked by arrows. Genome organization based on PiCV sequence MW656022 as representative.

Reference

[73] Khalifeh, A.; Kraberger, S.; Dziewulska, D.; Varsani, A.; Stenzel, T. A Pilot Study Investigating the Dynamics of Pigeon Circovirus Recombination in Domesticated Pigeons Housed in a Single Loft. *Viruses* **2021**, *13*, 964. https://doi.org/10.3390/v13060964.
